# Concentric and Eccentric Endurance Exercise Reverse Hallmarks of T-Cell Senescence in Pre-diabetic Subjects

**DOI:** 10.3389/fphys.2019.00684

**Published:** 2019-06-04

**Authors:** Marc Philippe, Hannes Gatterer, Martin Burtscher, Birgit Weinberger, Michael Keller, Beatrix Grubeck-Loebenstein, Johannes Fleckenstein, Katharina Alack, Karsten Krüger

**Affiliations:** ^1^Department of Sports Medicine, Institute of Sports Sciences, Giessen University, Giessen, Germany; ^2^Department of Sport Science, Medical Section, University of Innsbruck, Innsbruck, Austria; ^3^Department of Health Promotion and Prevention, Swiss Lung Association, St. Gallen, Switzerland; ^4^Institute of Mountain Emergency Medicine, EURAC Research, Bozen, Italy; ^5^Institute for Biomedical Aging Research, University of Innsbruck, Innsbruck, Austria; ^6^Department Exercise and Health, Institute of Sports Science, Leibniz University Hannover, Hanover, Germany

**Keywords:** TEMRA cells, naïve T-cells, eccentric exercise, concentric exercise, inflammation

## Abstract

The peripheral T-cell pool undergoes a striking age associated remodeling which is accelerated by progressive insulin resistance. Exercise training is known to delay several aspects of T-cell senescence. The purpose of the current study was to investigate the effect of 3 weeks regular concentric or eccentric endurance exercise training on the composition of the T-cell compartment in pre-diabetic subjects. Sixteen male older adults with impaired glucose tolerance were recruited and performed either concentric exercise (CE) or eccentric exercise (EE) walking 3 times a week for 3 weeks. Fasting venous blood sampling was performed before training and after the training intervention. Various T-cell subpopulations were analyzed by flow cytometry. We did not find significant time × group effects (interaction) but found several significant time effects for cell type ratios and cell subsets proportions. There was an increase of the CD4^+^/CD8^+^ (0.55 ± 0.85%; *p* = 0.033) and CD4^+^/CD3^+^ ratio (5.63 ± 8.44%; *p* = 0.018) and a decrease of the CD8^+^/CD3^+^ ratio (-0.95 ± 1.64%; *p* = 0.049) after training. We found proportional increases of CD4^+^/CCR7^+^/CD45RO^+^ central memory cells (5.02 ± 7.68%; *p* = 0.030), naïve CD8^+^/CCR7^+^/CD45RO^-^ (3.00 ± 6.68%; *p* = 0.047) and CD8^+^/CCR7^+^/CD45RO^+^ central memory cells (3.01 ± 3.70%; *p* = 0.009), while proportions of CD4^+^/CCR7^-^/CD45RO^-^ TEMRA cells (-2.17 ± 4.66%; *p* = 0.012), CD8^+^/CCR7^-^/CD45RO^-^ TEMRA cells (-5.11 ± 7.02%; *p* = 0.018) and CD16^+^ cells (-4.67 ± 6.45%; *p* = 0.016) decreased after training. 3 weeks of either CE or EE were effective in reversing hallmarks of T-cell senescence in pre-diabetic subjects. It is suggested that exercise stimulates production and mobilization of naïve T-cells, while differentiated TEMRA cells might disappear by apoptosis.

## Introduction

Due to increases in human life expectancy, aging is regarded as one of the biggest health issues worldwide. During aging, several functions of the immune system experience dramatic changes, which fundamentally affect health and survival. This process termed “immunosenescence” is complex and affects almost most cellular and humoral components of immunity. Clinically, these changes have noticeable consequences for the effectiveness of immune responses, the vulnerability to infection, the efficacy of vaccination, and reactivation of latent viruses in aging individuals. Together, these progressive deficits result in increased morbidity and mortality (Castle, 2000; Pawelec et al., 2010).

In particular, the T-cell pool undergoes a striking age-associated remodeling process. While peripheral cell numbers do not change, the relative composition of T-cell subpopulations is importantly affected. A major component of this change is represented by an inverted CD4^+^/CD8^+^ T-cell ratio which appears in parallel to a decline in naïve T-cell numbers and the accumulation of highly differentiated memory cells (Pawelec et al., 2010). Mostly, these cells are CD8-positive T-cells which lack the expression of the lymphoid homing receptor CCR7 and costimulatory molecules such as CD28 alongside re-expressing CD45RA, termed terminally differentiated effector memory (TEMRA) cells (Weinberger et al., 2007; Arnold et al., 2011). TEMRA cells are known to produce large amounts of pro-inflammatory cytokines suggesting that they are involved in the development of a systemic low grade inflammation during aging. The reasons for the progressive appearance of these cells are suggested to be the persistent exposure to pathogens, frequent reactivations of latent viruses, and increased levels of oxidative stress (Effros et al., 2003; Simpson et al., 2016). The concomitant increase of proinflammatory molecules during aging, particularly TNF-α (Bruunsgaard et al., 2003) and IL-6 (Cohen et al., 1997), is suggested to amplify T-cell differentiation and senescence (Di Mitri et al., 2011). Accordingly, critical age related T-cells changes are accelerated by the existence of metabolic risk factors like insulin resistance or type 2 diabetes (Ventura et al., 2017). In the end, TEMRA cells may show an impaired immune function due to a series of molecular changes and loss of co-stimulatory molecules (Goronzy et al., 2012). The general importance of the age associated changes in the T-cell repertoire is highlighted by their inclusion in the IRP. The IRP represents a cluster of immunological parameters, which was shown to be associated with a poor immune function in elderly and predictive of earlier mortality (Wikby et al., 1998).

Regular exercise has been shown to significantly improve several aspects of immune function that decline with ageing (Walsh et al., 2011). With regard to T-cells, exercise training in mice improved T-cell proliferation in response to an antigen encounter (Liu et al., 2017). In aging mice it was demonstrated that exercise restored the percentages of naïve and memory cells in the spleen toward that of young mice (Woods et al., 2003). Untrained humans exhibited higher proportions of late-stage differentiated CD4^+^ and CD8^+^ cells and lower proportions of naïve CD8^+^ cells compared to trained individuals (Brown et al., 2014). Therefore, it is proposed that regular training might delay the onset of T-cell ageing or even has rejuvenating effects. While in most therapeutic interventions concentric endurance exercise is used to stimulate patients’ immune system, eccentric endurance exercise has also been shown to affect T-cell immunity and metabolisms (Jensen and Richter, 2012; Peake et al., 2015). Compared to concentric endurance exercises such as uphill walking, eccentric endurance exercises such as downhill walking induce higher strain on the muscle while the metabolic cost remains relatively low (Johnson et al., 2002; Camillo et al., 2015). Thus, especially physically unfit subjects that one can find in a population of middle aged men suffering from pre-diabetes, could profit from an exercise form like downhill walking that is less demanding for the cardiovascular system than uphill walking (Drexel et al., 2008; Philippe et al., 2017).

The aim of the current study was to investigate the effects of a 3 weeks regular concentric (uphill walking) and eccentric (downhill walking) endurance exercise training in a real world setting (e.g., summer holidays in the Alps), on the composition of the T-cell compartment in pre-diabetic subjects. We hypothesize that exercise training induces an increase of naïve T-cells in parallel to a decrease of cells with a senescent phenotype. We further hypothesized that concentric and EE exhibit different effects.

## Materials and Methods

### Study Participants

Data were collected in the course of the interventional study performed by Philippe et al. (2017). Therefore, the additional analysis performed for the current study are described extensively, whereas a more detailed study protocol description can be found elsewhere (Philippe et al., 2017).

For this study, the same 16 male community-dwelling older adults with IGT (age: 57.0 ± 5.2 years, BMI: 28.1 ± 2.2 kg⋅m^-2^, body mass: 86.2 ± 10.2 kg, fasting plasma glucose: 6.8 ± 1.32 mmol⋅l^-1^) were analyzed. All subjects had an IFG [defined as: IFG: fasting plasma glucose 100 mg⋅dl^-1^ (5.6 mmol⋅l^-1^) to 125 mg dl^-1^ (6.9 mmol⋅l^-1^)] and/or IGT [IGT: 2-h plasma glucose in the 75-g oral glucose tolerance test 140 mg⋅dl-1 (7.8 mmol⋅l^-1^) to 199 mg⋅dl^-1^ (11.0 mmol⋅l^-1^)] and did not receive any glucose metabolism relevant medication. Exclusion criteria were acute or chronic diseases, smoking, and a BMI >30 kg⋅m^-2^. Subjects were randomly assigned to the CE (= uphill walking, *N* = 8) or the EE (= downhill walking, *N* = 8) group. All participants gave their written informed consent to participate in the study, which was performed according to the declaration of Helsinki and approved by the ethics committee of the Medical University of Innsbruck (Protocol ID: AN5029; ClinicalTrials.gov ID: NCT01890876).

### Exercise Protocol and Blood Sampling

A group of 8 participants (4 CE, 4 EE) performed the exercise protocol in spring 2013 and a group with another 8 participants in fall 2013 (4 CE, 4 EE). The training consisted of nine uphill walking exercise sessions for the CE group and nine downhill walking sessions for the EE group, which were performed on Mondays, Wednesdays, and Fridays on 3 consecutive weeks. The starting point for the CE group was 850 m above sea level and the finish point 1360 m above sea level. Both groups were brought to their starting point by car. During each uphill walking session 510 high meters and a horizontal distance of 5 km had to be covered at a pace perceived as somewhat hard [= Borg 13], without exceeding a RPE of 15 on the 6–20 Borg scale (Borg, 1970). The EE group walked the same path as the CE group but in the opposite direction. The EE participants were asked to walk as fast as possible or at a maximum intensity corresponding to RPE 15 but without running. The total estimated energy expenditure of the 9 walking sessions was 20785 ± 4232 kJ for the CE group and 10593 ± 2211 kJ for the EE group (Philippe et al., 2017).

Fasting venous blood sampling was performed at the same time of day, before training and 1–2 days after the last walking session.

### Analysis of Plasma Cytokines and Cytomegalovirus (CMV) Serostatus

The concentrations of TNF-α (TNF-α) and IL-6 in plasma were analyzed by using commercially available ELISA kits (R&D systems, Minneapolis, MN, United States). CMV serostatus was analyzed using a specific ELISA against CMV-IgG antibodies using a commercially available kit (Siemens, Germany).

### Analysis of T-Cell Subpopulations

**FIGURE 1:**
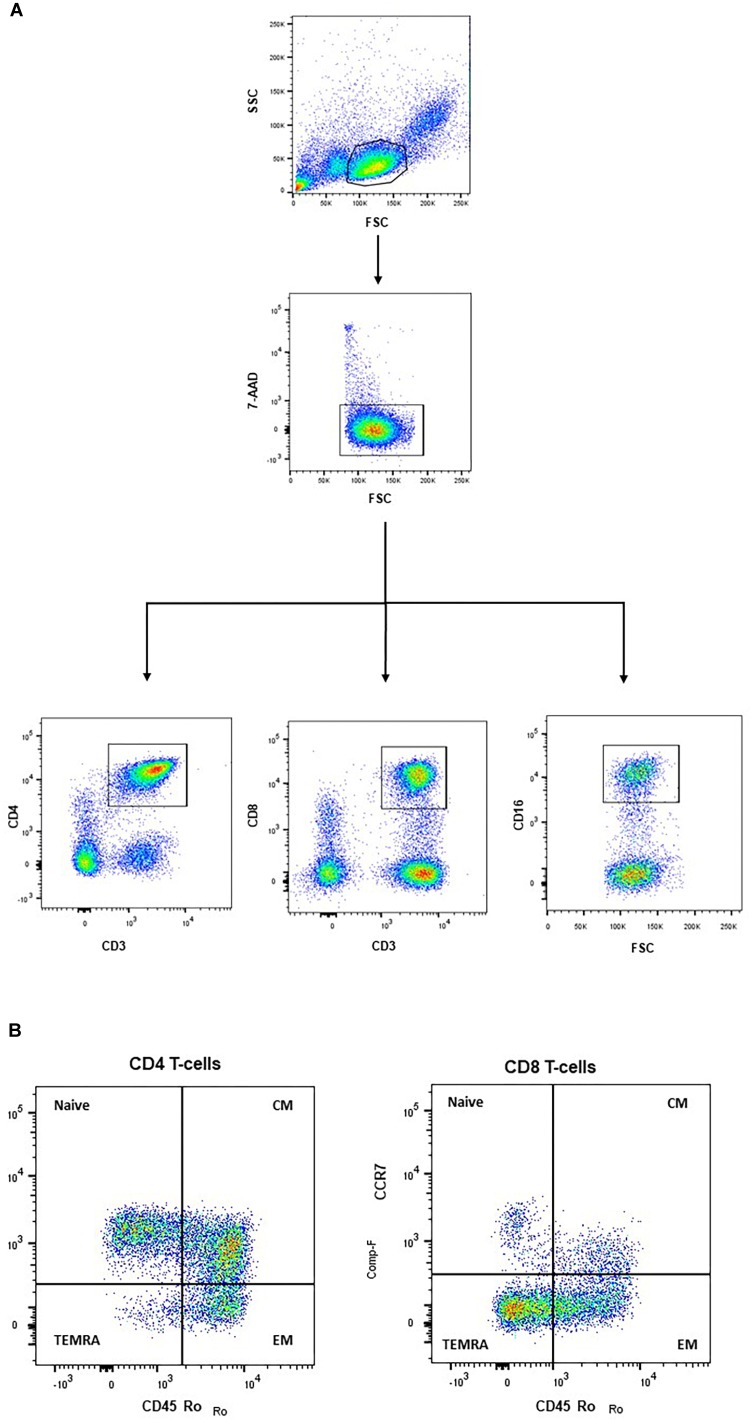
Frozen PBMCs were defrosted and stained with fluorochrome-conjugated antibodies. Gating strategy: Lymphocytes were gated in a FSC/SSC-dot plot and dead cells excluded using 7-AAD. From the living cell population, CD4 T-cells, CD8 T-cells and CD16^+^ cells were gated according to their specific surface markers **(A)**. T-cell subpopulations, within CD4 and CD8 T-cells, respectively, were defined by staining CD45RO and CCR7. NAIVE (CD45RO-/CCR7+): naive T-cells; CM (CD45RO+/CCR7+): central memory; EM (CD45RO+/CCR7-): effector memory; TEMRA (CD45RO-/CCR7-): effector memory + RA **(B)**.

PBMCs (peripheral blood mononuclear cells) were isolated by density gradient centrifugation using Ficoll paque (VWR, Austria) and frozen (-196°C) for analysis at a later time point. Analysis of T-cell subpopulations was performed by flow cytometry (FACSCANTO II, BD, United States) using specific labeled antibodies against CD3 (APCCy7), CD4 (APC), CD8 (BV421 or PECy7), CD45RO (PE), CCR7 (FITC), CD16 (FITC), CD19 (PE), and CD25 (PE) all from BD/Europe, Biozym/Germany or eBioscience/Austria and the viability dye 7-AAD (Sigma/Germany). At first, lymphocytes were gated on a forward scatter/side scatter (FSC/SSC) dot plot following gating on living cells and CD3/CD4 or CD3/CD8. Combinations of surface markers were classified to define the respective immune cell subpopulations as stated in Table 1 and Figure 1. For quality control, we followed standard procedures of the lab, which includes determination of the % bright bead robust CV for the measurements according to the manufacturer’s instructions.

**Table 1:** Proportional change of T-cell subpopulations before and after CE training.

					ANOVA		ANCOVA
						
Cell type (ratio)	Before CE	After CE	Before EE	After EE	Main effect time *p*-value (effect size eta^2^)	Interaction time x group *p*-value (effect size eta^2^)	Group (effect size eta^2^)
CD4^+^/CD8^+^	1.86 ± 0.81	2.56 ± 1.22	2.81 ± 1.08	3.19 ± 1.39	0.033^∗^ (0.306)	0.491 (0.037)	0.723 (0.012)
CD4^+^/CD3^+^	38.40 ± 13.68	47.76 ± 10.71	43.63 ± 9.92	45.00 ± 9.99	0.018^∗^ (0.362)	0.064 (0.239)	0.599 (0.026)
CD8^+^/CD3^+^	23.19 ± 9.38	22.26 ± 8.76	16.93 ± 5.77	15.94 ± 6.05	0.049^∗^ (0.266)	0.946 (<0.001)	0.658 (0.017)
**Cell type (% of all lymphocytes)**						
CD4^+^/CCR7^+^/CD45RO^-^	43.78 ± 12.79	41.63 ± 12.02	52.56 ± 16.64	51.40 ± 11.94	0.499 (0.036)	0.836 (0.003)	0.373 (0.073)
CD4^+^/CCR7^+^/CD45RO^+^	27.83 ± 8.62	32.98 ± 12.26	31.63 ± 9.29	36.50 ± 5.78	0.030^∗^ (0.313)	0.947 (<0.001)	0.844 (0.003)
CD4^+^/CCR7^-^/CD45RO^+^	18.58 ± 6.36	18.55 ± 7.51	12.70 ± 8.24	10.20 ± 7.00	0.367 (0.630)	0.376 (0.061)	0.740 (0.010)
CD4^+^/CCR7^-^/CD45RO^-^	9.84 ± 12.36	6.83 ± 7.02	3.13 ± 1.81	1.91 ± 0.84	0.012^∗^ (0.186)	0.908 (0.040)	0.740 (0.010)
CD25^+^/CD4^+^	29.91 ± 8.05	33.34 ± 8.62	39.36 ± 8.80	37.56 ± 11.21	0.740 (0.009)	0.295 (0.084)	0.654 (0.019)
CD19^+^ lymphocytes	9.44 ± 4.72	16.34 ± 22.76	8.24 ± 5.87	6.86 ± 4.72	0.497 (0.036)	0.313 (0.078)	0.640 (0.021)
CD16^+^ (lymphocytes)	24.10 ± 14.86	17.60 ± 8.24	16.63 ± 12.38	14.06 ± 9.76	0.016^∗^ (0.369)	0.253 (0.099)	0.216 (0.136)
CD8^+^/ CCR7^+^/CD45RO^-^	19.69 ± 15.19	23.16 ± 16.66	28.69 ± 19.11	31.14 ± 18.38	0.047^∗^ (0.175)	0.817 (0.006)	0.482 (0.046)
CD8^+^/CCR7^+^/CD45RO^+^	5.61 ± 4.00	9.31 ± 5.83	8.86 ± 3.80	11.09 ± 7.04	0.009^∗^ (0.417)	0.463 (0.042)	0.315 (0.092)
CD8^+^/CCR7^-^/CD45RO^+^	30.15 ± 15.75	28.91 ± 15.86	26.90 ± 9.52	26.41 ± 8.41	0.654 (0.016)	0.844 (0.003)	0.156 (0.174)
CD8^+^/CCR7^-^/CD45RO^-^	44.58 ± 17.86	38.65 ± 14.57	35.53 ± 12.78	31.36 ± 10.10	0.018^∗^ (0.359)	0.647 (0.017)	0.726 (0.012)
CD25^+^/CD8^+^	5.50 ± 1.43	6.86 ± 3.39	10.94 ± 6.22	10.77 ± 3.96	0.383 (0.059)	0.265 (0.094)	0.009^∗^ (0.475)

*^∗^*p* < 0.05*

### Statistical Analysis

Analysis was performed with the SPSS statistical software package (ver. 23.0; SPSS Inc., Chicago, IL, United States). Metric variables were visually (boxplot and skewness) and mathematically (Kolmogorov-Smirnov test) assessed for normal distribution. Means and standard deviations (SD) were calculated as descriptive statistics. A few parameters were not normally distributed in the CE or in the EE group. As there were no outliers, a sensitivity analysis was performed (repeated measures ANOVA vs. Wilcoxon test; repeated measures ANOVA vs. Mann-Whitney *U*-test) for these parameters. The sensitivity analyses showed a difference for CD4^+^/CCR7^-^/CD45RO^-^ cells and CD8^+^/ CCR7^+^/CD45RO^-^ cells, therefore, we opted for non-parametric testing for these variables (Wilcoxon test for effects of training over time; Mann-Whitney *U*-test for time × group effects, using the mean differences of after exercise minus before exercise values). For all other variables, the sensitivity analyses showed no marked differences between parametric and non-parametric testing, thus we opted to stick with the more robust parametric tests (Field, 2011). The effect of training over time (before and after), as well as the time x group effect (interaction) for cytokines, T-cell subpopulation, CD19^+^ B lymphocytes and CD16^+^ cells were assessed by repeated measures ANOVA, with group as the between-subjects factor. In addition, effect sizes (partial eta squared, eta^2^) were also calculated. As several cell parameters were different at baseline when comparing CE with EE, we performed an ANCOVA for all parameters with the pretest values as a covariate, to test, whether there was a difference in the post-test means by group after adjusting for the pretest observation. Relations between CMV serostatus and T-cell subpopulations before and after exercise as well as relations between changes of plasma cytokine levels and changes of T-cell subpopulation, CD19^+^ B lymphocytes and CD16^+^ cells were calculated with Pearson product-moment correlation coefficient. To assess whether there was a difference in the frequency of CMV-positive and CMV-negative participants between CE and EE, we performed a chi-squared test. Statistical significance level was set at *p* < 0.05.

## Results

We were not able to detect time x group (interaction) effects for any of the investigated parameters, i.e., the analysis of plasma cytokines (Table 2), and the analysis of T-cell subpopulations.However, time effects on cell type ratios and cell subsets proportions could be observed in both groups following eccentric and concentric training:

**Table 2:** Cytokine levels before and after CE and EE training.

					ANOVA	ANCOVA
					
Cytokine levels	Before CE	After CE	Before EE	After EE	ANOVA Main effect time *p*-value (effect size eta^2^)	ANCOVA Group (effect size eta^2^)
TNF-α (pg⋅ml^-1^)	1.99 ± 1.16	1.77 ± 0.60	1.43 ± 0.37	1.59 ± 0.68	0.862 (0.002)	0.760 (0.007)
IL-6 (pg⋅ml^-1^)	2.06 ± 0.90	1.83 ± 1.77	1.84 ± 1.24	2.84 ± 2.72	0.490 (0.035)	0.317 (0.077)

*^∗^*p* < 0.05*

The ratios of CD4^+^/CD8^+^ (*F*_(1,13)_ = 5.725; *p* = 0.033) and CD4^+^/CD3^+^ (*F*_(1,13)_ = 7.725; *p* = 0.018) significantly increased after training, while the CD8^+^/CD3^+^ ratio (*F*_(1,13)_ = 6.815; *p* = 0.049) significantly decreased after training (Table 2).

We found a significant increase of the relative proportion of CD4^+^/CCR7^+^/CD45RO^+^ central memory cells (*F*_(1,13)_ = 5.912; *p* = 0.030) and a significant decrease of CD4^+^/CCR7^-^/CD45RO^-^ TEMRA cells (*p* = 0.012) and the relative proportion of CD16^+^ cells (*F*_(1,13)_ = 7.617; *p* = 0.016) after training (Table 1).

Training significantly increased the relative proportion of CD8^+^/CCR7^+^/CD45RO^+^ central memory cells (*F*_(1,13)_ = 9.282; *p* = 0.009) and CD8^+^/ CCR7^+^/CD45RO^-^ naïve cells (*p* = 0.047), and significantly decreased the relative proportion of CD8^+^/CCR7^-^/CD45RO^-^ TEMRA cells (*F*_(1,13)_ = 7.286; *p* = 0.018; Table 1).

ANCOVA showed no significant group effect for the above mentioned parameter thus, we may assume that baseline differences between groups may not account for the observed time effects.

At baseline, 9 participants were CMV-positive and 7 participants were CMV-negative. Chi-squared test showed no difference in CMV serostatus (positive or negative) between CE and EE (*p* = 0.131). CMV serostatus neither correlated with any T-cell subpopulation before exercise nor with exercise induced changes of T-cell subpopulations. Changes of TNF-α plasma levels significantly positively correlated with changes of the CD16^+^ cells proportion (*R* = 0.533; *p* = 0.041).

## Discussion

The current study demonstrates that 3 weeks of concentric or eccentric endurance training were able to increase both, the CD4^+^/CD8^+^ ratio as well as the CD4^+^/CD3^+^ ratio in pre-diabetic subjects, indicating a relative increase in CD4^+^ T helper cells. The proportional increase of naïve and of central memory CD8^+^ cells after CE and EE indicates a shift to lower differentiated cell types. In parallel, the proportion of CD8^+^ TEMRA cells decreased indicating a relative reduction of highly differentiated T-cells. Surprisingly, despite CE and EE being different training modalities, inducing different stress on the contractile filaments of muscle and the muscle metabolism (Camillo et al., 2015; Philippe et al., 2017), we found no interaction effects regarding changes of cytokine plasma levels, cell ratios or CD4^+^ and CD8^+^ T-cell subset proportions.

A decreased CD4^+^/CD8^+^ ratio is an accepted hallmark of immunological ageing. In a series of longitudinal studies of octogenarians in exceptional health, and nonagenarians, this parameter was identified as part of the “Immune Risk Profile” (IRP) (Pawelec et al., 2010). Accordingly, it represents an important characteristic which predicts a greater all-cause mortality (Wikby et al., 1998). Chronic diseases which are accompanied by a systemic low grade inflammation, have been shown to amplify the decrease of the CD4^+^/CD8^+^ ratio, resulting in a compromised host protection (Richard et al., 2017). The current data demonstrate that 3 weeks of regular CE or EE were followed by an increase of the CD4^+^/CD8^+^ ratio. The concomitant increase of the CD4^+^/CD3^+^ ratio indicates that these changes are due to an increase of CD4^+^ T helper cells. The mechanisms for these changes are speculative. On the one hand, each bout of acute exercise mobilizes hematopoietic stem cells into blood which might travel to the thymus or extrathymic places for differentiation into naïve CD4^+^ cells (Krüger et al., 2015). On the other hand, exercise might mobilize more differentiated T-cells from the marginal pool and the spleen into the circulation (Krüger et al., 2008). Both mechanisms might contribute to altered CD4^+^ cell numbers. Regarding the slight decrease of the CD8/CD3 ratio, it is suggested that the relative number of CD8^+^ T-cells is lower after training. This assumption is supported by previous data from longitudinal training studies which have also reported declines in total CD8^+^ T-cells after 12 weeks and 6 months of endurance training (Baj et al., 1994; Gleeson et al., 2000).

Besides a general increase of CD4^+^ T helper cells, we also found changes in CD4^+^ T-cell subsets. While the proportion of CD4^+^ central memory cells increased, there was a decrease of CD4^+^ cells with TEMRA cell characteristics after training, without differences between CE and EE. These alterations represent a shift from more differentiated to less differentiated cells in the CD4^+^ T helper cell compartment. However, actually the concept of the CD4^+^ equivalent to TEMRA cells is intensively discussed and not clearly defined. Therefore, it is difficult to conclude any immunological impact of changes in this subpopulation (Larbi and Fulop, 2014).

With regard to CD8^+^ cells, changes in T-cell subsets were found after training, without differences between CE and EE. For naïve and central memory CD8^+^ T-cells, a proportional increase was found. Both cell types are known to be effective in response to invading antigens by having a high capacity to expand and proliferate (Sallusto et al., 2004). With regard to immunosenescence these changes seem to be important because decreased numbers of naïve CD8 T-cells represent an important biomarker of an ageing immune system (Appay and Sauce, 2014). The age-related reduction of naïve CD8 cells is suggested to be due to reduced T-cell maturation as a result of thymic involution, but also to reduced generation of lymphoid progenitors and to a higher susceptibility to death receptor-mediated apoptosis, triggered by peripheral inflammatory cytokines like TNF-α (Messaoudi et al., 2004; Emmons et al., 2017). Exercise is suggested to effectively affect all these processes. Accordingly, each acute bout of exercise mobilizes hematopoietic progenitor cells into blood (Krüger et al., 2015). It was further shown that T-cells of trained subjects exhibit an increased resistance to apoptosis (Krüger and Mooren, 2014). Finally, exercise training has anti-inflammatory properties resulting in reduced systemic TNF-α levels (Pedersen and Bruunsgaard, 2003). However, since we did not find a significant reduction of either, TNF-α or IL-6 after training, this mechanism is unlikely.

After CE and EE, a decrease of CD4^+^/CCR7^-^/CD45RO^-^ and CD8^+^/CCR7^-^/CD45RO^-^ cells was found, implicating a proportional reduction of TEMRA cells. An accumulation of TEMRA cells, specifically CD8^+^ T-cells, is an important feature of immune aging since these cells produce large amounts of pro-inflammatory cytokines after activation (Zhang et al., 2001). In addition, CD8 TEMRA cells have only a reduced capacity to proliferate, suggesting that an increase of these cells reduces an organism’s capacity to defend itself against invading pathogens. The age-related increase of TEMRA cells is even more pronounced in the T-cell pool of CMV-seropositive elderly compared to CMV-seronegative elderly persons (Simpson et al., 2016). Therefore, the CMV serostatus was analyzed, but could be excluded as a significant confounder in the present study. The reduced proportion of CD8^+^TEMRA cells after exercise is speculated to be the result of at least two different processes. On the one hand, the relative increase of other CD8 positive T-cell subpopulations might reduce the relative proportion of TEMRA cells. On the other hand, it was shown that acute bouts of exercise induces apoptosis in T-cells which was more frequently observed in more differentiated cells (Krüger et al., 2016). Integrating the exercise-induced increase of naïve T-cells and decrease of TEMRA T-cells in a holistic model, there might exist a negative feedback loop between cell death and mobilization of progenitor cells. Such a connection has been demonstrated in mice, where application of apoptotic bodies evoked the mobilization of hematopoietic progenitors. Accordingly, the loss of senescent cells by apoptosis might create space for the expansion of naïve T-cells (Simpson, 2011; Mooren and Krüger, 2015). However, since we did not analyze either apoptosis or progenitor cell mobilization, these mechanisms have to be addressed in future projects.

CD16^+^ represent a subgroup of lymphocytes which include NK cells, gamma-delta T-cells and subsets of CD8^+^ T-cells. The correlation analyses showed a significant positive correlation between changes of TNF-α and changes of CD16^+^ cells. Thus, the participants with the highest reductions of CD16^+^ cells exhibited the most important decreases of TNF-α plasma levels. While a direct link between these processes is speculative, both processes might reflect the anti-inflammatory effect of exercise training. Consequently, especially persons suffering from pre-diabetes may benefit from these alterations as TNF-α mediated low-grade inflammation is co-responsible for the development of insulin resistance (Pedersen, 2017).

Despite the lower estimated energy expenditure of the EE group compared to the CE group all observed effects were not different between CE and EE. It has previously been shown that despite the lower energy cost, EE had equal or even superior positive effects on glucose and lipid metabolism and inflammation (Drexel et al., 2008; Paschalis et al., 2010). Our results indicate that these findings may also be true regarding positive alterations of immunosenescence. Thus, uphill and downhill walking may equally be recommended for older persons and especially for subjects suffering from pre-diabetes.

### Limitations

The relatively small sample size may be considered as a limitation. Furthermore, the present investigation misses a control group. Yet, as the main aim of this study was to compare the effects of CE and EE on the composition of the T-cell compartment this limitation might be considered minor. Furthermore, we neither measured CD56^+^ surface marker in order to specifically characterize NK cells and neither monitored diet and caloric intake nor physical activity patterns during the study phase. Although we explicitly asked the study participants not to change their eating, drinking or physical activity habits during the study period, we cannot entirely rule out that additional life style changes might have influenced our results. In contrast to studies displaying indoor or laboratory training regimens, outdoor training cannot be comparably standardized. For example, participants had to encounter changing weather conditions, also due to different seasons. These uncontrollable external factors may have influenced the results.

## Conclusion

In conclusion, the current data implicate that 3 weeks of either concentric or eccentric endurance training increases the proportion of CD4^+^ T-cells and of naïve CD8^+^ T-cells and central memory CD4^+^ and CD8^+^ T-cells, and reduces the proportion of CD4^+^ and CD8^+^ TEMRA cells in pre-diabetic subjects. These changes might be favorable for patients since they represent an opposing trend against immunosenescence and might stimulate host defense against invading pathogens.

## Ethics Statement

All participants gave their written informed consent to participate in the study, which was performed according to the declaration of Helsinki and approved by the ethics committee of the Medical University of Innsbruck (Protocol ID: AN5029; ClinicalTrials.gov ID: NCT01890876).

## Author Contributions

MP, MB, and BG-L conceived and designed the research. MP, HG, BW, MB, and MK performed the experiments. MP, HG, MK, BW, and KK analyzed the data. KK and MP drafted the manuscript. MP, HG, MK, KA, JF, MB, BG-L, and KK edited and revised the manuscript. MP, HG, MK, BW, KA, JF, MB, BG-L, and KK approved final version of the manuscript.

## Conflict of Interest Statement

The authors declare that the research was conducted in the absence of any commercial or financial relationships that could be construed as a potential conflict of interest.

## Abbreviations

ANCOVA: analysis of covariance
ANOVA: analysis of variance
CE: concentric exercise
CMV: cytomegalovirus
EE: eccentric exercise
IFG: impaired fasting glucose
IGT: impaired glucose tolerance
IL-6: interleukin 6
IRP: immune risk phenotype
RPE: rate of perceived exertion
TEMRA: terminally differentiated effector memory cells
TNF-α: tumor necrosis factor-α
